# The biological function of the cellular prion protein: an update

**DOI:** 10.1186/s12915-017-0375-5

**Published:** 2017-05-02

**Authors:** Marie-Angela Wulf, Assunta Senatore, Adriano Aguzzi

**Affiliations:** Institute of Neuropathology, University of Zurich, Rämistrasse 100, CH-8091 Zürich, Switzerland

## Abstract

The misfolding of the cellular prion protein (PrP^C^) causes fatal neurodegenerative diseases. Yet PrP^C^ is highly conserved in mammals, suggesting that it exerts beneficial functions preventing its evolutionary elimination. Ablation of PrP^C^ in mice results in well-defined structural and functional alterations in the peripheral nervous system. Many additional phenotypes were ascribed to the lack of PrP^C^, but some of these were found to arise from genetic artifacts of the underlying mouse models. Here, we revisit the proposed physiological roles of PrP^C^ in the central and peripheral nervous systems and highlight the need for their critical reassessment using new, rigorously controlled animal models.

The cellular prion protein (PrP^C^) is a cell surface protein expressed in a variety of different organs and tissues with high expression levels in the central and peripheral nervous systems [1]. It is mainly known for its infamous role in prion diseases, where its misfolding and aggregation cause inevitably fatal neurodegenerative conditions [2]. Prion diseases are transmissible and misfolded prion protein (PrP^Sc^) is—according to the “protein-only hypothesis’”—the only disease-causing agent [3]. Under this view, it is puzzling that a protein underlying such severe diseases is highly conserved throughout mammals [4]. This suggests the existence of distinct benefits and, potentially, important physiological functions.

A definitive, fully satisfactory understanding of the physiological function of PrP^C^ has been lacking for a long time. Very recently, we identified a native function of PrP^C^ in the peripheral nervous system and the underlying mechanism of that function [5]. However, PrP^C^ is also highly expressed in the central nervous system (CNS) and its biological activity there is still far from being clear. This review will focus on the proposed roles of cellular prion protein in the central and peripheral nervous systems.

## The prion protein undergoes post-translational proteolytic processing

**Fig. 1:**
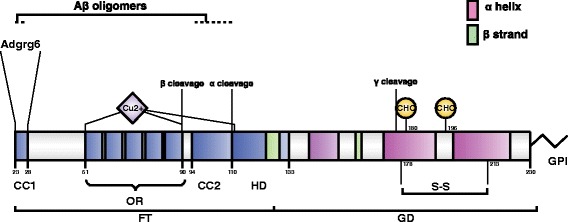
Structural organization of PrP^C^. Schematic representation of mature mouse PrP^C^, showing protein domains, sites of post-translational modification, and binding sites for divalent cations and protein interactors of functional relevance. *CC1* charge cluster 1, *OR* octapeptide repeats, *CC2* charge cluster 2, *HD* hydrophobic domain, *FT* flexible tail, *GD* globular domain. Structurally defined domains are depicted by *pink* (α-helix) and *green* (β-strand) *boxes. GPI* glycosylphosphatidylinositol anchor, *CHO* glycosylation site, *S-S* disulfide bridge. α, β, and γ cleavage sites are indicated. Copper binding sites (*Cu* ^*2+*^) within and outside the octapeptide region are reported as well as the sites involved in the interaction with Aβ oligomers and with the G-protein-coupled receptor Adgrg6

The cellular prion protein is encoded by the *Prnp* gene. In mice, the entire protein-coding open-reading frame is encoded within the third exon of *Prnp* [6–8]. After translation and cotranslational extrusion into the lumen of the endoplasmic reticulum, PrP^C^ adopts its physiological structure with a C-terminal globular domain and an N-terminal flexible tail [9] (Fig. 1). The N-terminal tail consists of two charged clusters (CC1 and CC2), the octarepeat region (OR) and a hydrophobic domain (HD). Additionally, two N-glycosylation sites are located in the globular domain upstream of the sialylated GPI-anchor at the C-terminus [10, 11].

After being transported to the cell membrane, PrP^C^ resides extracellularly in lipid rafts, where it is attached to the outer leaflet by a glycosyl phosphoinosityl (GPI) anchor [12]. It undergoes rapid constitutive endocytosis and subsequently either recycling or degradation [13, 14]. PrP^C^ endocytosis can occur by both clathrin-dependent [15] and caveolin pathways [16].

As part of its post-translational metabolism, PrP^C^ can undergo proteolytic cleavage events termed, in analogy to amyloid precursor protein processing, α-, β-, and possibly also γ-cleavage (Fig. 1). These cleavage events release the so-called N1 + C1, N2 + C2, and C3 fragments, respectively [17–20]. These events may be important for both physiology and pathology. Alpha cleavage prevents the C1 fragment from being converted into PrP^Sc^ [21], the rate of the beta cleavage is increased in the disease [19], and PrP^C^ deletion mutants lacking the alpha-cleavage site show spontaneous neurodegeneration exhibiting pathological features distinct from those of prion diseases (reviewed in [22]).

The enzyme responsible for PrP^C^ cleavage may not be unique, and several members of the ADAM (a_disintegrin and metalloproteinase) family have been implicated [23–26]. PrP^C^ can bind divalent cations such as copper and zinc [27] by the octarepeat-containing flexible tail and it has been reported to interact with a plethora of different proteins. These interactions have been taken to reflect its putative role in several cellular processes, but they may also simply be a consequence of the unstructured, flexible conformation of the N-terminus of PrP^C^.

## Genetic pitfalls of PrP^C^ gene ablation

Soon after PrP^Sc^ was proposed to be the causative agent of prion diseases, *Prnp* knockout mice lacking PrP^C^ were generated in order to answer the question whether the loss of physiological PrP^C^ function would lead to neurodegeneration in prion diseases. The first *Prnp* null mouse strain, designated *Prnp* ^-/-^, or Zurich I (ZrchI, *Prnp* ^ZH1/ZH1^), was produced in a mixed C57BL/6 J × 129/Sv(ev) background [28] and a second line of PrP^C^-deficient mice, known as Npu or Edinburgh (Edbg), was produced with a pure 129/Ola genetic background [29]. In a first round of characterization, these mice were not found to show any clear abnormality except for their resistance to prion infection [30]. They developed and bred normally, and although they displayed subtle alterations in behavior [28], their otherwise apparent normality seemed to rule out a physiological function of PrP^C^ that is essential for life. If there is one, it is highly redundant or it can be compensated for. PrP^C^-deficient mice from which the entire *Prnp* gene was removed [31–34] develop progressive cerebellar ataxia, which was originally attributed to the loss of PrP^C^ but was later discovered to be due to the deletion of a splice acceptor site in exon 3 of *Prnp* [35]. This led to aberrant overexpression of the PrP^C^ paralogue gene (*Prnd*) encoding Doppel (Dpl) [36, 37], causing selective neurodegeneration of cerebellar Purkinje cells. Notably, the reintroduction of *Prnp* in mice overexpressing *Prnd* in the brain rescued the phenotype, suggesting a functional link between the two proteins [38].

Later, using the Cre-loxP system, conditional PrP^C^ knockout NFH-Cre/tg37 mice were generated to examine the effects of acute PrP^C^ depletion on neuronal viability and function in the brain of 9-week-old adults. This approach was thought to avoid compensatory mechanisms active at the embryonal stage that would have masked PrP^C^ loss of function phenotypes [39]. Again, depleting neuronal PrP^C^ in adult mice did not result in neurodegeneration or histopathological changes, but it led to subtle electrophysiological abnormalities in the hippocampus (Table 1). A closer look at different neuronal and other cell functions in PrP^C^-ablated mice revealed a number of differences from wild-type mice that were attributed to the physiological function of PrP^C^. While some of these studies were consistent among different PrP^C^-deficient lines, others yielded contradictory results depending on methodologies and the mouse models that were used (Table 2).

**Table 1:** Lines of PrP^C^-ablated mice covered in this review

Name	Year produced	Reference	Genetic background	“Doppel artifact“
Zurich I (ZrchI, ZH1)	1992	[28]	**Mixed C57BL/6J x 129/Sv(ev)**	No
Edinburgh (Edbg)	1994	[29]	*129/Ola*	No
Nagasaki (Ngsk)	1996	[31]	**Mixed C57BL/6J x 129/Sv(ev)**	Yes
Rcm0	1997	[33]	**Mixed C57BL/6J x 129/Sv(ev)**	Yes
Zurich II (ZrchII, ZH2)	2001	[34]	**Mixed C57BL/6J x 129/Sv(ev)**	Yes
NFH-Cre/tg37 (adult onset)	2002	[39]	**Mixed C57BL/6J x 129/Sv(ev)**	No
Zurich III (ZrchIII, ZH3)	2016	[42]	*C57BL/6J*	No

*Bold* indicates mixed genetic background of at least two distinct mouse strains, possibly leading to the “flanking-gene problem”. *Italic* indicates mice maintained on single, pure genetic background

**Table 2:** Proposed physiological roles of cellular prion protein

Role of PrP^C^ in	Phenotype of *Prnp* ^*-/-*^ model system	Report (mouse model/cell line used)	Contradictory reports
Synaptic transmission and plasticity	Reduced long-term potentiation	[58] **(ZH1)** [29] (*Edgb*)	[61] (**ZH1**, **other** ^*a*^) [98] (**ZH1**)
	Reduced excitatory and inhibitory synaptic transmission	[58] (**ZH1**) [62] (**ZH1**, **Ngsk**)	[61] (**ZH1**, **other** ^*a*^) [147] (**ZH1**)
Memory formation	Reduced spatial learning and memory	[64] (**other** ^*b*^)	[28] (**ZH1**)
	Reduced avoidance learning and memory	[65] (**ZH1**) [148] (**Ngsk**)	[68] (**ZH1**)
Stabilization of sleep and circadian rhythm	Altered circadian rhythm, increased sleep fragmentation, increased SWA after sleep deprivation	[71] (**ZH1**, *Edgb*)	[74] (**Other** ^*b*^)
Neuronal excitability	Reduced Kv4.2 currents	[77] (**ZH1**, HEK293T)	
	Reduced sAHP and calcium-activated potassium currents	[79] (**ZH1**) [80] (**ZH1**) [82] (**ZH1**) [39] (**Tg35**) [83] (**ZH1**)	
	Increased susceptibility to Kainate-induced seizures	[84] (**ZH1**)	[41] (**Other** ^*b*^)
Calcium homeostasis	Reduced VGCC currents	[80] (**ZH1**)	[83] (**ZH1**)
	Increased calcium buffering	[83] (**ZH1**)	
Glutamate receptor function	Increased NMDA currents, nociception and depressive-like behavior	[90] (**ZH1**) [91, 92] (**ZH1**)	
	Upregulation of Kainate receptor subunits	[84] (**ZH1**)	
Neurite outgrowth	Delayed development of cerebellar circuitry	[120] (**ZH1**)	
	Reduced neurite outgrowth in vitro	[106] (**ZH1**)	
Toxicity elicited by oligomeric species	Protected from LTP reduction induced by toxic Aβ species	[98] (**ZH1**, **other** ^*c*^)	[102] (**ZH1**) [103] (**ZH1**) [104] (**Other** ^*b*^) [105] (**Other** ^*a*^)
Neuroprotection	Larger lesions in model of acute cerebral ischemia	[122] (**ZH1**) [123] (**ZH1**) [124] (**ZH1**)	
	Decreased SOD activity	[133] (**ZH1**)	[135] (**ZH1**)
Copper, zinc, iron, and lactate metabolism	Reduced zinc content in primary neurons	[95] (**ZH1**, SH-SY5Y)	
	Increased lactate-uptake in cultured astrocytes	[96] (**ZH1**)	
	Altered iron and copper metabolism	[139] (**Other** ^a^)	
Peripheral myelin maintenance	Age-dependent demyelinating neuropathy	[141] (**ZH1**, *Edgb*) [42] (*ZH3*)	

*Bold* indicates mixed genetic background of at least two distinct mouse strains, possibly leading to the “flanking-gene problem”. *Italic* indicates mice maintained on single, pure genetic background. Mouse lines specified as “other” are: ^a^ZH1 backcrossed to FVB; ^b^Edgb (back-)crossed to C57BL/10; ^c^Edbg backcrossed to C57BL6

A genetic confounder has been shown to underlie some of these inconsistencies [40, 41]. For many years, knockout alleles were usually created in embryonic stem cells from the Sv129 strain of mice, and the resulting mice were backcrossed to C57BL/6 mice [42]. This practice typically leads to variable, poorly controlled Mendelian segregation of polymorphic alleles whose distribution depends on their genetic linkage to the knockout allele. All *Prnp* knockout mouse lines have been generated in this way with the exception of the “Edinburgh” mouse, which was maintained in a pure 129 background [42]. Even after more than 12 generations of backcrossing, a small part of the chromosome around the *Prnp* locus still stems from the 129 strain, raising the question whether any observed phenotypes were actually due to polymorphisms in genes flanking *Prnp*. Indeed, we found that SIRPα, a polymorphic *Prnp*-flanking gene, is actually responsible for an alleged *Prnp* ^*-/-*^ phenotype: the inhibition of macrophage phagocytosis of apoptotic cells that was observed in PrP^C^-deficient mice with mixed genetic background but not in co-isogenic *Prnp* ^*-/-*^ mice [40]. Recently, a new PrP^C^-deficient mouse strain, *Prnp* ^ZH3/ZH3^, was produced in our lab using TALEN-mediated genome editing in fertilized mouse oocytes and maintained in a pure C57BL/6 J genetic background [42]. These strictly co-isogenic C57BL/6 J-*Prnp* ^ZH3/ZH3^ mice differ from wild-type mice only by eight deleted nucleotides in the *Prnp* reading frame. In an effort to improve the quality of studies on the function of the cellular prion protein, we are distributing *Prnp* ^ZH3/ZH3^ mice without requesting any kind of Material Transfer Agreement, hence enabling better-controlled future studies. In view of the broad availability of *Prnp* ^ZH3/ZH3^ mice, we contend that the use of mixed-background PrP^C^-deficient mice is obsolete and liable to artifacts.

Do further mammal species teach us more about the function of PrP^C^? The gene encoding PrP^C^ has been ablated experimentally in cattle [43] and goats [44], and a naturally occurring *Prnp* knockout goat has been reported [45]. While no pathological phenotypes were reported in any of these animals, it may be rewarding to perform specific investigations of these animals, e.g., concerning the integrity of the peripheral nervous system in advanced age.

A large set of human genomic data was analyzed to quantify the penetrance of variants of the human PrP^C^ gene (*PRNP*) in prion disease [46]. Surprisingly, heterozygous loss-of-function variants were identified in three individuals. These individuals in their 50s and 70s are probably healthy, and no evidence of any neurological defect or peripheral neuropathy was documented. This result suggests that heterozygous loss of *PRNP* in humans may not be haploinsufficient. It remains to be assessed, however, whether homozygous deletion and therefore complete loss of PrP^C^ may create a disease in humans.

## Evidence for a role of synaptic PrP^C^ in memory and sleep

PrP^C^ is strongly expressed in both neurons and glial cells of the CNS [1]. In neurons, PrP^C^ is preferentially localized in the pre- and postsynaptic compartments of nerve terminals. Immunocytochemical studies by light and electron microscopy in primate and rodent brains [47, 48], as well as examination of an EGFP-tagged PrP^C^ in transgenic mice, showed that PrP^C^ is enriched along axons and in pre-synaptic terminals [49, 50] and that it undergoes anterograde and retrograde axonal transport [51, 52]. PrP^C^ is also present in postsynaptic structures [53, 54]. It has recently been shown that sialic acid within the GPI-anchor is important for targeting PrP^C^ to synapses [55]. This expression pattern implies that PrP^C^ might be involved in preserving normal synaptic structure and function by regulating synaptic transmission and plasticity (Fig. 3). Supporting this notion, synaptic dysfunction and synaptic loss are a prominent and early event in prion diseases [56, 57].

Early reports showed that PrP^C^-deficient mice (both ZH1 and Edbg) display reduced long-term potentiation (LTP) in hippocampal Schaffer collaterals and weakened inhibitory GABAergic synaptic transmission [58, 59]. These defects have been claimed to be rescued by a human *PRNP* transgene [60]. However, none of these results were reproduced in PrP^C^-deficient mice of three different genetic backgrounds (Table 2). These discrepancies have remained essentially unexplained for the past 20 years [61]. Later studies reported a PrP^C^ dosage-dependent facilitation of synaptic transmission, with PrP^C^-over-expressing mice exhibiting supra-physiological synaptic transmission [62]. This effect seemed to result from a more efficient recruitment of pre-synaptic fibers that, in turn, may depend on PrP^C^ expression levels.

LTP is one of the neurophysiological correlates of synaptic plasticity, the ability of synapses to change their strength in response to previous activity. Because synaptic plasticity (LTP) in the hippocampus underlies learning and memory formation [63], any LTP deficits may result in cognitive defects. Initial reports did not show any reduction in memory performance of *Prnp* ^ZH1/ZH1^ mice in the Morris Water Maze test [28]. However, a later study found deficiencies in spatial learning and memory in PrP^C^-deficient mouse lines on various backgrounds [64]. These deficits were explained by reduced LTP in the dentate gyrus in PrP^C^-deficient mice in vivo*,* and were rescued by neuronal expression of PrP^C^. *Prnp* ^ZH1/ZH1^ mice were reported to have impaired memory performance only when aged (9 months). Several molecular mechanisms for this defect have been proposed, but none were verified at the mechanistic level [65–67]. These results stand in contrast to another study carried out in *Prnp* ^ZH1/ZH1^ mice that did not reveal any memory impairment up to an age of 2 years [68]. Thus, a role for PrP^c^ in memory is still contentious.

A role of PrP^C^ in sleep homeostasis and sleep continuity has also been proposed and loss of such a function for PrP^C^ would be of clinical relevance. Indeed, it would explain the disruption of the sleep pattern that occurs as a prominent symptom in some forms of prion diseases like sporadic and familial fatal insomnia [69, 70]. Both *Prnp* ^ZH1/ZH1^ and co-isogenic *Prn*p^Edbg/Edbg^ mice were reported to display altered circadian rhythms, increased sleep fragmentation, and increased slow wave activity (SWA) following sleep deprivation [71]. The latter phenotype was rescued by reintroduction of PrP^C^ expression [72]. It was suggested that the difference in SWA between wild-type and *Prnp* null mice reflected a function of PrP^C^ in neurotransmission or a protective role on synapses [73]. Another study confirmed sleep disturbance in *Prnp* knockout mice. However, using a prolonged sleep deprivation protocol, the defect was found to be associated with reduced slow-wave activity and to altered hormonal reactivity to prolonged stress [74]. Interestingly, deterioration of slow-wave activity was found to contribute to sleep deficits in Alzheimer’s disease (AD) and was reversed by enhancing GABA_A_ergic inhibition [75].

The molecular bases of sleep regulation are not completely understood. Tantalizingly, recent work indicates that calcium-dependent hyperpolarization is critical to sleep duration, and that sleep deterioration is associated with impairment of calcium-dependent potassium channels, voltage-gated calcium channels (VGCC), and N-methyl-D-aspartate (NMDA) glutamate receptors [76]. Since both hyperpolarization linked to calcium dyshomeostasis and NMDA receptor-related hyperexcitability were documented in *Prnp* ablated mice (discussed in the next section), loss of PrP^C^-dependent control of these ion channels may underlie the sleep disruption in PrP^C^-deficient mice and perhaps also in prion diseases.

## Possible functions for PrP^C^ are suggested by interaction partners

**Fig. 2:**
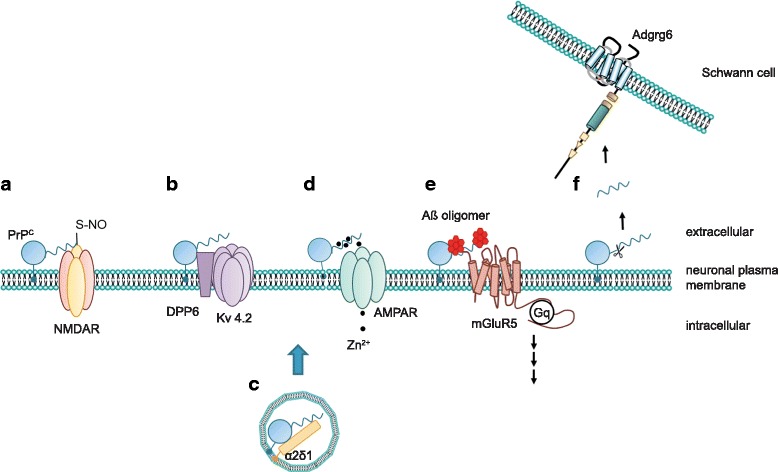
PrP^C^ exerts its functions via distinct mechanisms. The cellular prion protein may utilize several mechanisms to modulate cellular functions. As schematically depicted in **a**, PrP^C^ may directly alter the function of its target protein by mediating posttranslational modifications, for example, by promoting the S-nitrosylation of the NMDA receptor. Alternatively, PrP^C^ modulates auxiliary proteins of ion channels, thereby regulating the biophysical properties of the channel (**b**) or its trafficking (**c**). Another function of PrP^C^ arises from its ability to bind divalent cations such as zinc (Zn^2+^) or copper (Cu^2+^). It was claimed that PrP^C^ may buffer these cations within the synaptic cleft and may facilitate their uptake (**d**) via AMPA receptors. Some better-defined actions of PrP^C^ include its binding to misfolded oligomeric protein species and signaling in complex with other membrane receptors (**e**). Additionally, PrP^C^ can signal in *trans* by its N-terminal cleavage products, which may bind to other receptors, prominently including the G-protein-coupled receptor Adgrg6 (**f**)

Albeit controversial, the participation of PrP^C^ in neurobiological processes, and particularly in sleep regulation and memory, raises the question whether the cellular prion protein modulates synaptic mechanisms and neuronal excitability at a molecular level. Insights into possible mechanisms may be provided by the documented interaction of PrP^C^ with several ion channels and metabotropic glutamate receptors (Fig. 2). However, caution is needed while screening the crowded PrP^C^ interactome: only interactions with molecular partners displaying a functional correlate to PrP^C^ binding should be considered of potential biological relevance.

**Fig. 3:**
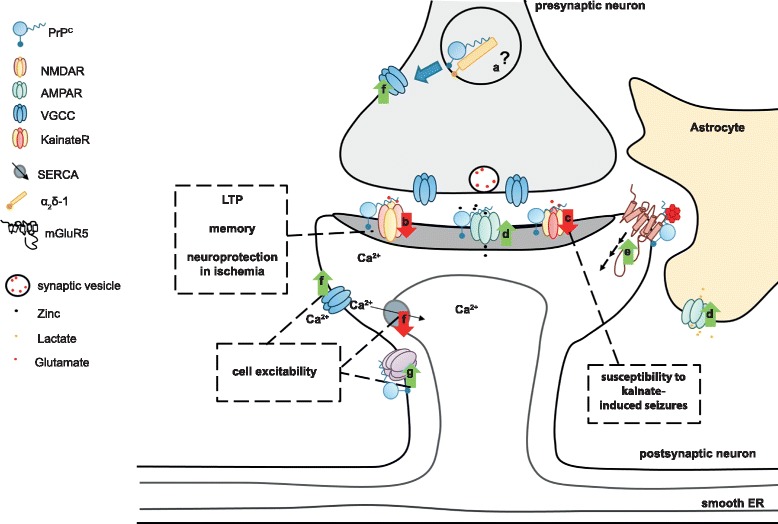
Schematic overview of possible physiological functions of PrP^C^ and their effect in the central nervous system. PrP^C^ regulates ion channels and neurotransmitter receptors at the pre- and postsynaptic levels. **a** PrP^C^ might modulate VGCC trafficking at the presynapse via interaction with the α_2_δ-1 VGCC subunit. **b** Postsynaptically, PrP^C^ dampens NMDA receptor-mediated currents by modulating various receptor subunits of this channel. It was speculated that control of NMDA receptor function might be related to certain reported phenotypes of PrP^C^-ablated mice. **c** PrP^C^ may also control the glutamatergic system by modulating the subunit composition of kainate receptors. This possibly relates to increased susceptibility of PrP^C^-ablated mice to kainate-induced seizures. **d** PrP^C^ associates with, and promotes cell surface localization of, AMPA receptor subunits. This facilitates zinc uptake at the synaptic cleft via AMPA receptors. On astrocytes, a PrP^C^–AMPA complex may be involved in the uptake of lactate. **e** PrP^C^ binds to toxic oligomeric protein species. PrP^C^ binds to Aβ oligomers and, in complex with metabotropic glutamate receptor 5 (mGluR5), was proposed to trigger intracellular signaling related to Alzheimer's disease pathology. **f** PrP^C^ controls calcium influx via interaction with different ion channels. Additionally, PrP^C^ was claimed to regulate calcium storage via the sarcoplasmic/endoplasmic reticulum calcium ATPase (SERCA). **g** PrP^C^ positively modulates potassium currents as exemplified by association with DPP6, an auxiliary subunit of the Kv 4.2 potassium channel. Control of calcium and potassium channels might be related to the alleged function of PrP^C^ in neuronal excitability. *ER* endoplasmic reticulum

PrP^C^ interacts with Dipeptidyl peptidase-like 6 (DPP6), an auxiliary subunit of the voltage-gated potassium channel 4.2 (Kv4.2), which leads to increased and prolonged currents through this channel, thereby reducing cellular excitability [77]. Also, PrP^C^ and a mutant PrP^C^ version that is linked to a genetic prion disease have been shown to co-immunoprecipitate with the auxiliary subunit α2δ-1 of VGCCs in transgenic mice [78]. Mutant PrP^C^ affected α2δ-1 trafficking and function at the synapses. The molecular role of PrP^C^ in VGCC function under physiological conditions remains unclear (Fig. 3). However, deficits in VGCC currents and calcium homeostasis were reported in *Prnp* ^ZH1/ZH1^ neurons [79, 80] and were proposed to underlie the reduced slow after hyperpolarization (sAHP) seen in PrP^C^-deficient mice. The sAHP is a property of many neurons that is evoked by repetitive action potentials and controls subsequent action potential firing. The intermediate-conductance, calcium-activated potassium channel (IkCa) has been claimed to control this neurophysiological parameter [81].

Not only could the sAHP defect be reproduced by independent research groups in *Prnp* ^ZH1/ZH1^ mice [80, 82, 83], it has also been shown in an adult-onset model of PrP^C^ depletion, indicating that this is not a developmental phenotype [39]. At the cellular level, reduced sAHP is expected to result in increased neuronal firing. Increased neuronal excitability in the PrP^C^-deficient hippocampus has also been reported in the context of higher vulnerability of PrP^C^-deficient mice to kainate-induced seizures (Fig. 3). This was related to the interaction of PrP^C^ with the kainate receptor subunit GluR6/7 [84]. Additionally, in the absence of PrP^C^, GluR6/7-containing KA receptors were upregulated and the neurotoxic signaling was enhanced [84–86]. This suggests a neuroprotective role of PrP^C^ against excitotoxic insults.

However, whether this represents a bona fide PrP^C^ phenotype remains controversial. *Prnp* ^-/-^ mice on a different genetic background displayed a reduced susceptibility to kainate-induced seizures in the absence of PrP^C^. As this phenotype could not be rescued by reintroduction of an exogenous *Prnp* gene, it is suspected that polymorphisms in some unidentified *Prnp*-flanking genes may underlie the discrepant phenotypes [41]. More recently, however, although the effect of the “*Prnp*-flanking gene” in the KA-mediated responses was confirmed in PrP^C^-deficient mice of a mixed background (B6129 and B6.129), enhanced sensitivity to epileptogenic drugs was found in the co-isogenic *Prnp* ^Edbg/Edbg^ mouse [87]. The molecular mechanism underlying this phenotype is unclear but the aa32–93 region of PrP^C^ (spanning the octapeptide repeats) and its glycosylphosphoinositol anchor may be involved. Consistent with the higher neuronal excitability of PrP^C^-deficient mice, anatomical changes within the hippocampus indicated a reorganization of neuronal circuitry similar to the “epileptic neuronal network” seen in certain human epilepsies [88].

PrP^C^ might also act as a modulator of glutamate receptors of the NMDA subtype. These are heterotetramers composed of two GluN1 subunits and two GluN2 subunits of different subtypes [89]. It was found that PrP^C^ inhibited NMDA receptors and prevented potentially excitotoxic calcium influx through these channels by association with NMDA receptors containing the GluN2D subunit [90], as shown by co-immunoprecipitation and immunofluorescence imaging. Absence of PrP^C^ led to upregulation of GluN2D-containing NMDA receptors and enhanced signaling due to prolonged kinetics of NMDA-mediated currents [90]. This was subsequently linked to increased depressive-like behavior and increased nociception in PrP^C^-deficient mice. Both phenotypes were rescued by pharmacological inhibition of NMDA receptors [91, 92]. Additionally, copper-dependent interaction of PrP^C^ with the GluN1 subunit was documented, which was involved in nitrosylation of NMDA subunits GluN2A and GluN1. This represents a second mechanism by which the presence of PrP^C^ reduces NMDA currents and signaling [93, 94].

The putative control of ionotropic glutamate receptors by PrP^C^ may be even more complex, since PrP^C^ also interacts with α-amino-3-hydroxy-5-methyl-4-isoxazolepropionic acid (AMPA) receptor subunits GluA1 and GluA2. This interaction may be relevant to the PrP^C^-mediated cellular uptake of zinc through AMPA receptors [95] and to the regulation of lactate transport in astrocytes [96]. Whether the binding of PrP^C^ with AMPA receptor subunits also plays a role in AMPA receptor function remains to be elucidated.

## PrP^C^ interactions with metabotropic glutamate receptors and Aβ

Recently, PrP^C^ has been shown to interact not only with ionotropic but also with metabotropic glutamate receptors of group I, mGluR1 and mGluR5 (Fig. 2). Metabotropic glutamate receptors are members of the G protein-coupled receptor (GPCR) superfamily of seven transmembrane-domain proteins that are activated by glutamate and transduce intracellular signals via G proteins. PrP^C^ binds to and signals through mGluR5 in disease-related conditions [97].

Several studies found that Aβ oligomers, the neurotoxic protein species involved in AD, can bind to PrP^C^ [98, 99] (Figs. 1 and 2) and activate the Fyn kinase through mGluR5 [97]. Aβ–PrP^C^–mGluR5 complexes are responsible for facilitation of long-term depression (LTD) in vivo [100] and dendritic spine loss in cultured neurons [97]. The Aβ–PrP^C^–mGlu5R complex might act upstream of the phosphorylation of the NMDA receptor subunit GluN2B. This event within the pathogenic cascade triggered by Aβ oligomers requires PrP^C^-dependent Fyn activation [54] and underlies the Aβ oligomer-induced disruption of LTP in AD.

Focusing on the role of PrP^C^, two main considerations can be drawn from these studies. First, PrP^C^ appears to function as a cell surface receptor for synaptotoxic oligomers of the Aβ peptide and, as reported by Resenberger and colleagues [101], of other β-sheet-rich neurotoxic proteins. However, whereas the physical interaction of PrP^C^ with Aβ oligomers was confirmed [99, 102], it is unclear whether PrP^C^ is necessary for the synaptotoxic effect of Aβ oligomers [102–105]. Secondly, although the interplay between mGluR5 and PrP^C^ may be relevant to AD pathology, it remains unclear whether the binding to PrP^C^ affects physiological functions of group I metabotropic glutamate receptors. Intriguingly, a role for the PrP^C^–mGluR1 complex in neurite outgrowth has been reported [106].

## Role of PrP^C^ in development

During murine embryonic development, PrP^C^ is expressed as early as a few days post-implantation, suggesting a possible role in development [107, 108]. Transcriptomic analysis of PrP^C^ knockout embryos showed several differentially expressed genes (DEGs), while the number of DEGs in brains of adult PrP^C^-deficient mice is almost negligible [42, 109]. Interestingly, the number of DEGs is higher in brains where PrP^C^ had been knocked out postnatally, suggesting that the function of PrP^C^ may be compensated for by other proteins during development [110]. To date, only a few in vivo studies on the role of PrP^C^ in CNS development are available. They suggest that PrP^C^-ablated mice exhibit reduced proliferation rates of neuronal progenitor cells in the embryonic, newborn, and adult CNS [111, 112]. Additionally, increased proliferation of oligodendrocyte precursor cells with a concomitant maturation delay of oligodendrocytes and astrocytes has been reported [111, 113]. These observations were supported by the results of several in vitro experiments [111, 113, 114]. However, as the genetic background can influence brain development and adult neurogenesis [115], these studies should be confirmed in coisogenic PrP^C^-ablated mice.

Additionally, in vitro studies suggest that PrP^C^ is implicated in the regulation of neuritogenesis [116, 117] as well as axonal growth [48, 118, 119]. Also, there is some evidence that PrP^C^ is involved in the development of the cerebellar circuitry, leading to delayed motor development of PrP^C^-deficient mice [120].

## Possible neuroprotective roles of PrP^C^

PrP^C^ may have a neuroprotective role in a mouse model of cerebral ischemia, as PrP^C^-deficient mice show larger lesions in acute cerebral ischemia. Furthermore, overexpression of PrP^C^ can reduce the lesion size compared to wild-type mice [121–124]. Attenuation of NMDA signaling by PrP^C^ has been proposed to be the basis of a neuroprotective role of PrP^C^ against NMDA-mediated toxicity in ischemia [125]. Additionally, it was found that cleavage of PrP^C^ into its N- and C-terminal fragments is enhanced under ischemic conditions and these cleavage products can themselves be neuroprotective [124]. In particular, the N-terminal cleavage fragment (N1) might be neuroprotective against staurosporine-induced Caspase-3 activation in a model of pressure-induced ischemia in the rat retina [126]. These results are supported by several in vitro studies, where expression of PrP^C^ was protective against staurosporine or anisomycin-induced apoptosis [127, 128]. Conversely, loss of PrP^C^ was beneficial against glutamate-induced excitotoxicity in vitro, an effect supposedly mediated by increased uptake of glutamate in PrP^C^-ablated astrocytes [129].

The protective function of the N1 fragment is also very intriguing in the context of the Aβ oligomer-related synaptotoxicity. This intrinsically disordered N-terminal portion of PrP^C^ is involved in binding to β-sheet-rich peptides like Aβ oligomers [99, 101] and mediates the detrimental effects of Aβ oligomers on synaptic function as mentioned before. However, in its soluble form as secreted upon PrP^C^ cleavage, N1 acted in a decoy receptor-like mode: it prevented Aβ peptide fibrillization and reduced the neurotoxicity of amyloid-β oligomers in vitro and in vivo [130]. Additionally, the rate of PrP^C^ alpha-cleavage is increased in brain tissue from patients suffering from AD and it was proposed that alpha-cleavage represents an endogenous protective mechanism against amyloid-β toxicity in humans [131].

However, PrP^C^-deficient mice do not exhibit altered amyloid-β toxicity [102–105] and there was no protective effect of PrP^C^ in mouse models of other neurodegenerative diseases, including Parkinson's and Huntington's disease, as well as a mouse model of tauopathy [124, 132].

Based on in vitro studies, by virtue of its ability to bind copper, PrP^C^ has been proposed to participate in resistance to oxidative stress by preventing reactive oxygen species (ROS) generation via free copper-mediated redox reactions. Also, PrP^C^ was at some point thought to regulate the function of superoxide dismutase (SOD) [133]. It was even proposed that PrP^C^ could act as a SOD by itself [27, 134]. However, a function of PrP^C^ in copper metabolism is still controversial and the influence of PrP^C^ on either SOD level or the intrinsic dismutase activity of PrP^C^ was shown by us and others to be artifactual [135, 136]. There might be, however, alternative ways in which PrP^C^ protects against ROS toxicity. For instance, PrP^C^-dependent expression of antioxidant enzymes was suggested as an explanation for resistance to oxidative stress mediated by PrP^C^ [137, 138] as well as a conjectured PrP^C^ function in iron metabolism and control of redox-iron balance in cell lines [139, 140].

## Role of PrP^C^ in the peripheral nervous system

**Fig. 4:**
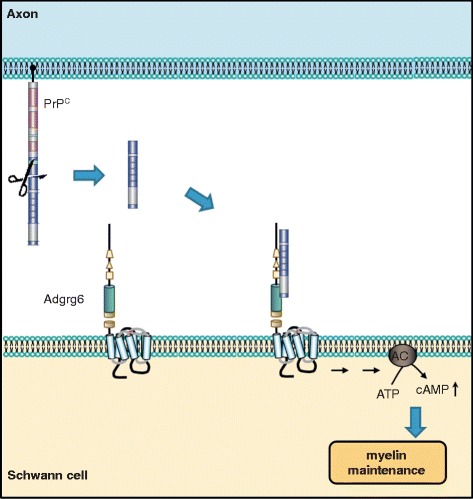
Axonal PrP^C^ promotes myelin maintenance in *trans* via Adgrg6 on Schwann cells. Mice devoid of PrP^C^ develop a chronic demyelinating neuropathy, which suggested a pro-myelinating function of PrP^C^. In the peripheral nervous system, the N1 fragment of axonal PrP^C^ interacts with Adgrg6 expressed on Schwann cells. This binding elicits activation of Adgrg6, which signals via adenylyl cyclase, thereby leading to increased cellular levels of cAMP. This triggers a well-defined downstream signaling cascade promoting myelin maintenance

PrP^C^-deficient mice of five different PrP^C^-knockout strains, including the *Prnp* ^ZH3/ZH3^ mice (coisogenic to BL/6 mice), develop a late-onset peripheral neuropathy, indicating that peripheral myelin maintenance is a bona fide physiological function of PrP^C^ [42, 141, 142]. PrP^C^ neuronal expression and amino-proximal cleavage (Fig. 2) are necessary for the promyelinating signal [141]. It was then discovered that the very N-terminal polycationic cluster of PrP^C^ binds to the G-protein-coupled receptor Adgrg6 (Gpr126) on Schwann cells (Fig. 1), eliciting a promyelinating cAMP response in vitro and in vivo in mice and zebrafish (Fig. 4) [5]. This pointed to the N-terminal fragment of PrP^C^ as a promyelinating factor that might serve as a possible treatment in other peripheral chronic demyelinating polyneuropathies.

In the CNS, Gpr126 is expressed by the Bergmann glia of the developing cerebellum, but not on mature oligodendrocytes, which are responsible for myelination [143]. However, some PrP^C^ deletion mutants lacking the cleavage site for the N-terminal fragment production also showed severe demyelination in both the spinal cord and cerebellar white matter in vivo [144, 145]. Thus, although in the CNS an eventual PrP^C^ function in myelin homeostasis is dispensable, a contribution of aberrant PrP^C^ function in demyelinating diseases in the brain is a conceivable scenario. Moreover, whether the N-terminal cleavage product of PrP^C^ is also signaling via other G-protein-coupled receptors in distinct biological processes is likely, but remains to be elucidated.

## PrP^C^ function: the next chapters

All available data point to PrP^C^ exerting its function in concert with additional membrane proteins. On one hand, PrP^C^ can regulate the cellular transport and localization of its binding partners. On the other hand, PrP^C^ can directly modulate the functionality of the binding partner—as seen for certain ion channels and ionotropic glutamate receptors. Also, PrP^C^ can signal in *trans* via its N-terminal cleavage products. Finally, PrP^C^ appears to scavenge amyloid aggregates of Aβ, and it will be interesting to see whether further pathological aggregates can also be recognized by PrP^C^. Given these findings, the question arises whether the cellular prion protein needs its misfolding-prone structure with a disordered flexible tail to fulfill its physiological function. The fact that the functional domains of PrP^C^ are conserved from avians to mammals speaks in favor of this hypothesis [4, 146]. Even though the interpretation of many studies is hampered by the genetic impurity of the mouse models used, there is enough evidence that PrP^C^ plays a role in several physiological functions in the central and peripheral nervous systems. Nevertheless, it appears implausible that PrP^C^ is involved in such a large number of cellular functions, particularly in view of the small number of validated pathological phenotypes in PrP^C^-deficient mice. The emergence of new, rigorously controlled animal models will be of help for revisiting and critically assessing some of these phenotypes.
